# Views of Service Users, Their Family or Carers, and Health Care Professionals on Telerehabilitation for People With Neurological Conditions in Ghana: Qualitative Study

**DOI:** 10.2196/49501

**Published:** 2024-03-27

**Authors:** Lorna Paul, Katie Thomson, Shadrack Osei Asibey, Marian Brady, Frederike van Wijck, Derrick Antwi, Eric Nkansah Opoku, Fred Stephen Sarfo

**Affiliations:** 1 School of Health and Life Sciences Glasgow Caledonian University Glasgow United Kingdom; 2 Department of Psychiatry Kwame Nkrumah University of Science and Technology Kumasi Ghana

**Keywords:** telerehabilitation, low- to middle-income country, LMIC, service user, health care professional, qualitative study, caregiver, neurorehabilitation, barriers, facilitators, eHealth, focus group, thematic analysis

## Abstract

**Background:**

Up to 50% of people in low- and middle-income countries do not receive the rehabilitation they require. Telerehabilitation has the potential to improve access to neurorehabilitation services especially in low- and middle-income countries. Although there are reports of the barriers and facilitators to telerehabilitation in such settings, almost all are anecdotal. Furthermore, family or carers have a significant influence on the adoption and success of telerehabilitation, but their views have not been reported.

**Objective:**

This study aimed to investigate the views of service users, their family or carers, and health care professionals (HCPs) on telerehabilitation for people with neurological conditions in Ghana.

**Methods:**

Two focus groups were held at Komfo Anokye Hospital in Kumasi, Ghana: one in person for service users (n=11) and their family or carers (n=9), conducted in the Ghanaian language of Twi, and one hybrid for HCPs (n=18) conducted in English. The mean (SD) age of the service users was 59.8 (8.6) years; 5 users had a stroke and 6 had Parkinson disease. The HCP group consisted of 7 speech and language therapists, 3 physiotherapists, 3 occupational therapists, 3 medical staff, 1 nurse, and 1 industry representative. Focus groups were semi-structured and explored previous experiences of telerehabilitation, perceived benefits and challenges, and solutions to overcome these challenges. Focus groups were audio transcribed, and the service user transcript was translated into English. The resulting transcripts were analyzed using thematic analysis.

**Results:**

Overall, participants were positive about the role of telerehabilitation but recommended hybrid delivery, with in-person rehabilitation in the early stages and telerehabilitation in the later stages. In relation to telerehabilitation in Ghana, there were 3 main themes: benefits, challenges or barriers, and implementation. Benefits included the convenience and lower cost for service users, the higher dose of therapy possible, and increased access for people in remote areas. However, challenges included lack of a stable internet connection, cost of phones and data packages, and low levels of literacy. Implementation issues included cultural relevance, information governance, and the platform used to deliver telerehabilitation, with most participants being familiar with WhatsApp.

**Conclusions:**

Telerehabilitation has the potential to be a useful method of delivering rehabilitation to people with neurological conditions in Ghana, especially in a hybrid rehabilitation model with telerehabilitation augmenting in-person sessions. However, many people were unaware of telerehabilitation, and challenges such as a reliable internet connection, cultural relevance, and costs need to be addressed. Clinical trials of low-cost telerehabilitation interventions contextualized to the specific user group are required.

## Introduction

The World Health Organization defines rehabilitation as “a set of interventions designed to optimize functioning and reduce disability in individuals with health conditions in interaction with their environment” [1]. They also highlight the significant unmet need for rehabilitation services that is most evident in some low- and middle-income countries (LMICs), where up to 50% of people do not receive the rehabilitation they require [1].

Rehabilitation in Ghana, an LMIC, is provided by the public and private sector and costs between US $5 and $20 per session. Rehabilitation sessions are paid for by the individual or through various national insurance schemes. Even where individuals have insurance, the coverage may be limited and “top-up payments” are required. The duration of neurological rehabilitation can vary between 3 and 12 months depending on the condition, and often, there is a waiting list of 1-2 months.

Numerous barriers to rehabilitation have been cited in LMICs, such as Ghana, including low numbers of therapists, especially in rural locations, services being concentrated in main towns or cities with the majority of the population living rurally, transport issues, costs for appointments, and lack of specialist staff and equipment [2-5].

As well as experiencing barriers to rehabilitation, LMICs are experiencing an increase in life expectancy and greater numbers of people with noncommunicable diseases including neurological conditions [2,6,7]. Although in its early stages, telemedicine is increasingly being explored in LMICs to deliver care to challenging or remote areas. Telerehabilitation, a branch of telemedicine, may provide part of the solution to the increased patient demand coupled with restricted service especially in neurological rehabilitation. Telerehabilitation is defined as the provision of rehabilitation, including physiotherapy, speech and language therapy, and occupational therapy, over distance and, oftentimes, using communication technology [8]. Potential telerehabilitation technologies include telephone and video calls, apps, virtual reality, and robotics [4]. A recent review suggests that the telephone or video is the main media through which telerehabilitation is delivered worldwide [9].

The evidence base for the acceptability, feasibility, and clinical and cost-effectiveness of telerehabilitation for people with neurological conditions is increasing. Several recent systematic and scoping reviews suggest that telerehabilitation improves access to services and is generally well received by patients and therapists, with high adherence to telerehabilitation programs and low adverse events, supporting its safety in practice [10,11]. Evidence of the clinical effectiveness of telerehabilitation for people with neurological conditions is mixed, but overall it is reported to be at least equivalent to standard care [12,13]. There is, however, limited evidence on the cost-effectiveness of telerehabilitation [6,9]. Much of the research in telerehabilitation has been undertaken in high-income countries, with notably fewer studies in LMICs [6,14,15], where the rehabilitation context, as well as the barriers and facilitators to the feasibility, adoption, scalability, and sustainability of telerehabilitation, may be quite different. In terms of neurological conditions, most telerehabilitation research in LMICs has been conducted in stroke [3,14,16].

To influence rehabilitation and improve patient outcomes, technology needs to be adopted into services. There are various models and theories of technology adoption including the Technology Acceptance Model and Self-Determination Theory. Central to these models are the beliefs and attitudes of the users of the technology. In the context of telerehabilitation, only a few previous studies exist on the views and beliefs of therapists and patients in LMICs and beyond [7]; however, these studies tend to be process evaluations of specific telerehabilitation interventions that are being researched and thus are open to selection bias as participants are exposed to the technology under study conditions. Furthermore, although telerehabilitation generally happens at home, there is a recognition that almost no research has sought the views of the patient, carer, or family member—although they have a strong and significant influence on the adoption and success, or otherwise, of telerehabilitation interventions [17].

Telerehabilitation is in its infancy in LMICs, including Ghana; however, to ensure successful adoption, implementation to routine practice, and scalability, it is important to understand the views of health care professionals (HCPs), service users (patients), and their carers. Therefore, the aims of this study were to investigate the views of HCPs involved in neurological rehabilitation, service users with a long-term neurological condition, and the carer or family member of someone with a long-term neurological condition in terms of previous experience of telerehabilitation, the perceived potential benefits, the potential challenges, and possible solutions to overcome these challenges in Ghana.

## Methods

### Study Design

Focus groups were used as the method of data collection for this qualitative study, and this paper is presented in line with the Consolidated Criteria for Reporting Qualitative Research guidelines. Separate focus groups were conducted: one for service users and carers or family members and another for HCPs and IT industry representatives. Both focus groups were conducted in person in Komfo Anokye Hospital in Kumasi, Ghana, with HCPs given the option of joining remotely via teleconference. The service user focus group was conducted in person to ensure that those who did not have technology or technology skills could contribute, to encourage engagement, and to support those with communication problems. For service users and carers traveling, expenses were covered and both groups were provided with lunch at the end of the focus group. The service user and carer focus group was conducted in Twi, the local language, and the HCP focus group was conducted in English.

### Inclusion Criteria

Inclusion criteria for the stakeholder group were HCPs (physiotherapists, occupational therapists or speech and language therapists, or medical staff) with experience of working in neurological rehabilitation in Ghana or staff with expertise in commissioning or delivering health services (commissioner or industry expert) and able to speak and understand English and attend the focus group either in person or via teleconference (Zoom).

Inclusion criteria for the service users were people with a neurological condition such as stroke, Parkinson disease (PD), or spinal cord injury and able to travel to the venue. Carers or family members had to have experience of caring for someone with a neurological condition and able to travel to the venue.

### Recruitment

A convenience sample of service users, who fulfilled the inclusion and exclusion criteria, was identified from two sources: (1) members of a support group for people with PD were telephoned by the research coordinator (SOA) and (2) stroke survivors attending a neurology clinic at Komfo Anokye Hospital 2 weeks before the focus groups were approached by the research coordinator. The study was explained to potential participants, and if they were happy to take part, they were given the details of the focus group date, time, and venue. They were given the option of bringing a carer or family member to the focus group although that was not a requirement. A convenience sample of HCPs was recruited from the professional networks of the research team. They were contacted through email or WhatsApp groups and asked to take part. If they were interested, study information was emailed to them with details of the date, time, and venue of the focus group, or if attending via Zoom, a link was shared. Consent was taken from all participants at the start of the focus group.

### Focus Groups

A focus group schedule was prepared for each of the 2 focus groups including main questions and prompts. For both groups, the questions were related to their experiences of telerehabilitation, perceived potential benefits of delivering or receiving rehabilitation via telerehabilitation, perceived potential challenges or difficulties, and suggestions for overcoming these difficulties.

The service user and carer focus group was facilitated by the male research coordinator in the room (SOA). This facilitator had been involved in recruitment of participants, had experience of conducting focus groups, and spoke the local dialect (Twi). The HCP focus group was facilitated by a senior female researcher from the United Kingdom (LP) via Zoom. This facilitator was a physiotherapist, with experience in telerehabilitation research and facilitating focus groups. She did not know any of the participants except those within the research team. The research team was introduced, and participants were provided with background information on the purpose of the study. The service user focus group lasted approximately 1 hour and the HCP group 1.5 hours. Both were audio-recorded and transcribed verbatim in the language in which they were conducted. The transcript of the service user and carer focus group was translated into English by professional transcribers from the University of Energy Sunyani, Ghana, and checked by the research team for accuracy. In addition, research team members made field notes during and after the focus groups.

### Analysis

Thematic analysis was the method of data analysis using the 6 phases outlined by Braun and Clark [18], including data familiarization, generation of initial codes, generation of themes, review of themes, definition of themes, and writing the report. Initial coding of the transcripts was completed by one researcher (KT) who had no previous relationship with study participants and checked by a second researcher (LP) who had conducted the HCP focus groups. Themes were derived from the data, and initial themes were presented to the research team (in verbal, written, and diagrammatic form), which were further refined after feedback and discussion. Thematic analysis was undertaken using Microsoft Word (Microsoft Corporation) with documentation shared at each stage ensuring transparent recording of the data analysis process with the research team. Thematic analysis of each focus group took place separately before a final round of analysis integrated these together. Further feedback was provided by the research team before the final themes were confirmed.

### Ethical Considerations

Ethical approval was received from the Ethics Committee of Kwame Nkrumah University of Science and Technology, Kumasi, in December 2022 (reference CHRPE/AP/822/22), and all participant data were deidentified. Informed consent was provided by all participants. Travel expenses were covered and lunch was provided for participants who attended in person focus groups.

## Results

### Characteristics of Participants

A total of 13 service users agreed to take part in the focus group; however, 2 were unable to attend on the day of the focus group, so overall 11 service users (9 male users) took part (Table 1). Service users had a mean (SD) age of 59.8 (8.6) years; 6 lived in an urban location, and 5 in a semiurban location. Six service users had PD, and 5 had had a stroke. The mean (SD) age of those with stroke was 53 (8.3) years, and the mean (SD) time since stroke was 4 (1.6) years. In contrast, the mean (SD) age of those with PD was 65.5 (2.5) years, and they had had the condition for a mean (SD) of 3.8 (2.1) years. In addition, 9 carers took part (4 male carers), and their mean (SD) age was 38.4 (8.6) years. They had a variety of occupations and had been caring for people with PD (n=6) and stroke (n=3) for a mean (SD) of 3.4 (2.2) years (Table 1).

Service users had different prior exposure to telerehabilitation, both synchronous and asynchronous. One had participated in a previous trial of an app that delivered an individualized rehabilitation program remotely supervised by a therapist [14], some had received rehabilitation plans via WhatsApp to undertake without supervision, and others had no previous experience of telerehabilitation.

**Table 1:** Demographic details of service users (n=11) and carers (n=9).

Demographic					Value
**Service users (n=11)**					
	**Sex, n**				
			Male	9	
			Female	2	
	Age (years), mean (SD)				59.8 (8.6)
	**Diagnosis of service user, n (%)**				
			Stroke	6 (55)	
		Parkinson disease			5 (45)
	Time since diagnosis (years), mean (SD)				6.0 (3.9)
	**Residence, n (%)**				
			Urban	6 (55)	
			Semiurban	5 (45)	
			Rural	0 (0)	
**Carer or significant other (n=9)**					
	**Sex, n**				
			Male	4	
			Female	5	
	Age (years), mean (SD)				34.8 (8.6)
	**Diagnosis of service user, n (%)**				
			Stroke	3 (33)	
			Parkinson disease	6 (67)	
	Length of care (years), mean (SD)				3.3 (2.2)
	**Relationship to service user, n (%)**				
			Daughter	4 (44)	
			Brother	2 (22)	
			Spouse	1 (11)	
			Son	1 (11)	
			Unknown	1 (11)	
	**Occupation, n (%)**				
			Trader	2 (22)	
			Driver	2 (22)	
			Hairdresser	1 (11)	
			Caterer	1 (11)	
			Shoemaker	1 (11)	
			Seamstress	1 (11)	
			Student	1 (11)	
	**Residence, n (%)**				
			Urban	5 (56)	
			Semiurban	4 (44)	
			Rural	0 (0)	

Eighteen stakeholders took part in the focus groups (10 female and 8 male). In total, 7 speech and language therapists, 3 physiotherapists, 3 occupational therapists, 2 neurologists, and 1 specialist physician, 1 nurse, and 1 person from industry participated. Eleven of the participants were aged 30-39 years, 3 were aged 20-29 years, and 3 were aged over 40 years. They had been in their current post for a mean of 4.8 (SD 3.3) years, and all worked in urban locations, most in the 2 main cities of Accra and Kumasi.

**Figure 1:**
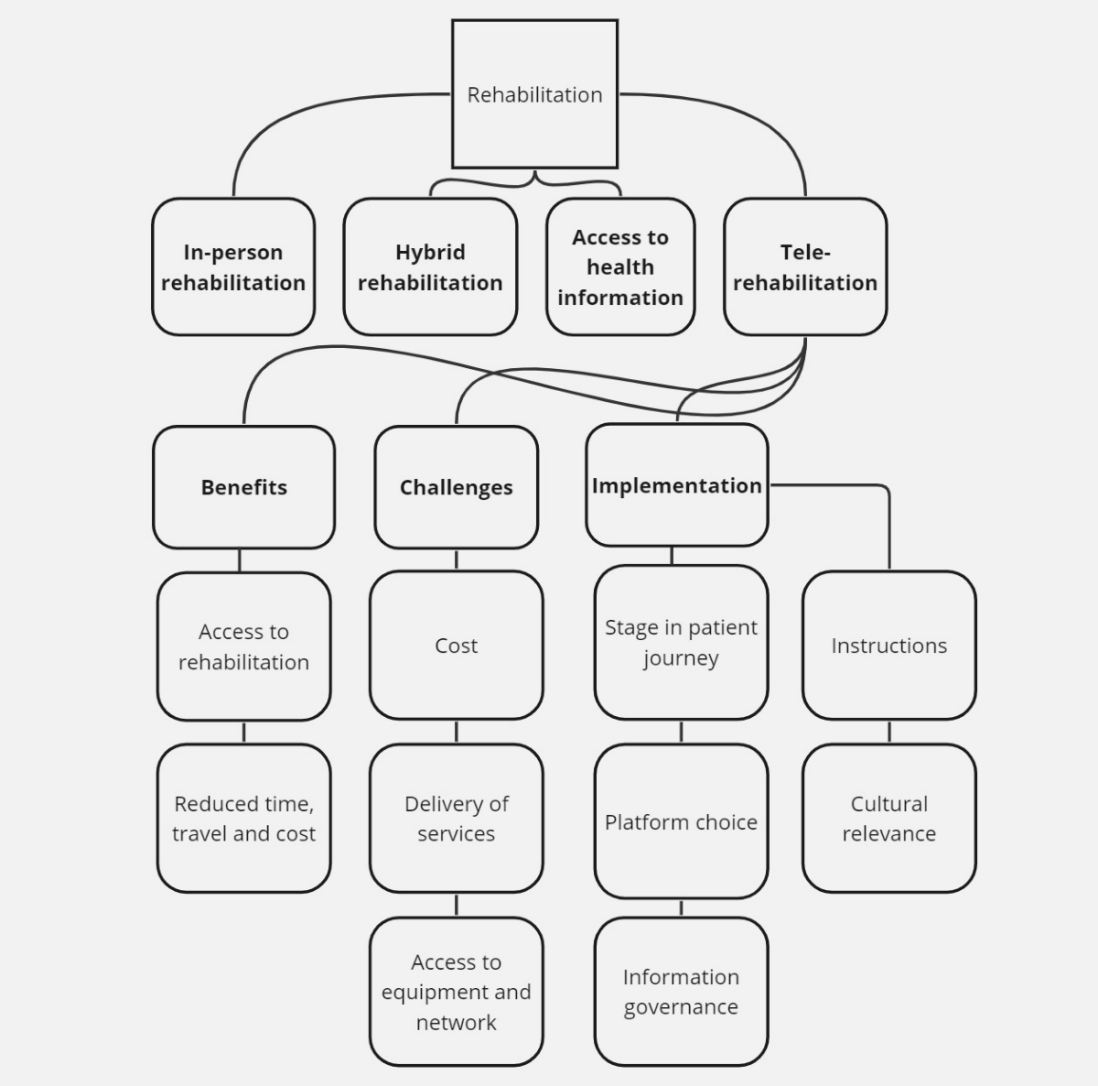
Focus group themes.

Analysis of the focus groups resulted in the following themes and subthemes (Figure 1).

### Rehabilitation Services

Service users identified different ways of accessing rehabilitation services, in-person services, telerehabilitation, and hybrid (a combination of in-person and remote services). Service users also expressed a need for increased awareness of the differing ways to access rehabilitation services and a need for reliable health information.

#### In-Person Rehabilitation

Service users valued in-person rehabilitation for the purpose of monitoring their rehabilitation progress and receiving feedback on this.

If there is an improvement, the doctor or the health facility that will tell you to either reduce or increase some aspect of your rehabilitation so that the body will recover.

Service user

It was however acknowledged that in-person rehabilitation had limitations particularly when there was a large demand for services, which could result in long queues at the health care facility, or the feedback received was not as hoped.

I stopped the rehab because of waking up early to join a queue.

Service user

One physio teasingly told me that my rehab has taken too long which downed my emotion and that made me stop coming for physio.

Service user

#### Hybrid Rehabilitation

Some service users and carers expressed a preference for a hybrid model of rehabilitation (in-person and telerehabilitation), which they had previously found useful.

I feel like if we combine the two it will help, both should go hand in hand.

Service user and carer

Both, because sometimes you may miss or do certain procedures wrongly without knowing but going to the hospital you will be able to know the exact procedure, then you can go ahead and practice at home.

Service user

#### Access to Health Information

A need for access to reliable health information was highlighted by both patients and carers with the potential for technology to be used as means of distributing information. Concern was expressed at misinformation, which hindered treatment such as medication use.

There should be associations to broadcast to people and to serve as guidance for others to follow.

Service user and carer

I have seen that everything is going on well here, but the information given is very low and because of that most people depend on herbal medicines.

Service user and carer

#### Telerehabilitation

Service users and carers who had experienced telerehabilitation found this to be a helpful way to access services and were happy to recommend this method of service delivery to others.

Yes, (I would recommend it) because I have tried and tested and it really helped me.

Service user and carer

We the caregivers also go through the same stress as the patients, so our experiences shows that the tele-rehab is very good.

Carer

For others, this was a novel way to access rehabilitation services, which, they thought, would be of benefit, indicating that they would be happy to try this in the future.

Gives me hope that even if a person gets any neurological disorder, the telerehab is there to help them get back their strength.

Service user and carer

However, there was a lack of awareness of telerehabilitation with service users, suggesting that further awareness raising was needed as well as ensuring that access was available throughout Ghana.

Many Ghanaians are not aware of the telerehab and so it should be publicised through TV and other media groups.

Service user and carer

Telerehabilitation should be extended to clinics to enhance easy access. It should not be in the big hospitals alone.

Service user and carer

A range of telerehabilitation services were described by service users who had experienced stroke (including those with aphasia), those with PD, and those with tinnitus or balance issues. These services included checking rehabilitation progress or receiving speech and language therapy sessions. In addition, service users sought further follow-up and reminders through telerehabilitation.

HCPs also had a range of experiences using telerehabilitation ranging from no involvement to using telerehabilitation for a range of rehabilitation purposes including teaching exercises, promoting engagement in activities, conducting hearing assessments, sending intervention messages and information, balance re-education, and reviewing videos to direct parent-led rehabilitation.

### Benefits of Telerehabilitation

#### Reduction in Travel, Time, and Associated Costs

A range of benefits were identified by service users, carers, and HCPs, including a reduction in travel, which made rehabilitation more convenient, easier to access, less stressful, accommodated other caring responsibilities, and was more affordable. Less time was also spent waiting for rehabilitation once patients and carers arrived at the rehabilitation unit.

It helped because having her come all the way to the clinic and the child who didn’t sit and fussy two hours was also time that was solved with tele-therapy.

HCP

It reduces financial costs to the hospital and the stress involved in sitting in a trotro (car).

Service user and carer

#### Increased Access to Rehabilitation

HCPs identified that they were able to access service users who lived some distance away (including visual access to the home environment) increasing access and relevance of the rehabilitation services delivered. This provided the opportunity for further rehabilitation, increasing the intensity, consistency, and adherence to rehabilitation offered.

with the in-person you’re scheduled to come let’s say two or three days a week but with telerehabilitation, even if you want to do it every day as far as the resources are there, you can do it every day. It also allows you to do it more frequently and over a more sustained period of time. That’s one of the benefits.

HCP

It can help with intensity because the more we meet them online we can achieve our goals. It is easy to find your therapy with more therapy sessions without having to move from one location to another. You sit in the comfort of your home and have more therapy sessions within the scheduled period but with a cost-effective system.

HCP

HCPs also saw the benefits of using devices such as mobile phones as a repository for information. This provided service users with a reminder of their rehabilitation exercises.

Most patients forget the therapy exercise we do for them so probably videoing it and saving for patients can help them do it at their leisure time.

HCP

HCPs reported that developing new ways of delivering rehabilitation services encouraged them to be creative in their approach including delivering content in a range of local dialects with the potential of developing new rehabilitation services or using technology to measure changes in service user knowledge for example.

I would like to add it makes you creative as a professional because you look for other means of making it fun with telerehabilitation in order to suit your client. It brings out creativity in you.

HCP

I think it will give the opportunity to assess the clients’ environment for recommendations for possible adaptations to enhance function because when the person comes in-person, they may describe the home environment and we don’t know exactly how it looks but through telerehab with video conference or picture you can see how the environment really looks like for recommendations for the adaptations.

HCP

#### Rehabilitation Progress Using Telerehabilitation

Service users highlighted the progress that they had made with their rehabilitation delivered using telerehabilitation, which included improvements in speech, arm movements, activity levels, and independence in activities.

It is through the tele-rehab that I am able to lift my hand today. It really helped me.

Service user

I’m forever grateful for the physios because up till date, they still call me and they helped in so many ways.

Service user

### Challenges of Telerehabilitation

Challenges of delivering telerehabilitation were identified by service users, carers, and HCPs including access to equipment and availability of a reliable internet connection, the cost of data packages, and challenges to rehabilitation delivery.

#### Equipment and Network Access

Availability of equipment and the requirement of a reliable network needed for telerehabilitation were highlighted as a barrier. Most service users in the focus group had access to mobile phones (including smartphones that access the internet), but not all did. Challenges with unreliable internet connections meant therapists often prepared alternatives such as printed exercise sheets, sending SMS text messages to promote engagement, watching videos offline, use of images (rather than videos), or switching platforms seeking a better connection. At times, however, the connection was so poor that therapy sessions were abandoned and rescheduled or therapists advised returning to in-person rehabilitation. For some service users, this meant finding other solutions including referral to local therapists to access rehabilitation.

Only 35% of our patients said that they own a smartphone and even of that 35% it’s not necessarily their own but there is a smartphone in the house. It’s not as if the smartphone belongs to them.

HCP

The network decided to fail all of us. When we call again, we can’t hear anything. We used other platforms FaceTime, other things, but the network just wouldn’t channel us until we had to put the whole therapy to an ending try to refer to someone closer to him.

HCP

In terms of connectivity, we can record the videos, store them, and send them so that when the connectivity improves the person can use the stored videos to be able look back on their exercise.

HCP

#### Financial Cost

Those who did have access to smartphones acknowledged that there was a financial burden of purchasing packages or “bundles” for calls and network access. Some rehabilitation services that required use of additional equipment provided this, but not all did.

Most of us are retired so money is hard to come by so it will really help us (if the equipment and data package are provided).

Service user

#### Rehabilitation Delivery

Use of telerehabilitation meant that therapists had to find new ways of delivering therapy services for an individual rather than their usual in-person service that included group therapy, acknowledging that not all service users had the digital literacy skills or ability needed to use technology.

because if I am at the physiotherapy department I can supervise maybe 4 or 5 patients simultaneously; this one is doing this, this one is doing that, I can just observe them but in telerehab they have to do one on one supervision so that may also eventually reduce the number of participants or patient they can attend to at a time if it is ongoing supervision they have to do.

HCP

Sometimes they are not very tech savvy so we need to see them in-person. And we have had some patients try it but most are not tech savvy so we haven’t been able to expand this to all our clients.

HCP

Another barrier I was thinking about was that if the client has multiple deficits so maybe visual needs and other possible deficits, I think that may impact teletherapy.

HCP

Other challenges with delivery of rehabilitation, such as location or timing of therapy sessions, the move toward carer involvement, and the type of therapy session being delivered, were highlighted.

Mum and I decided to have teletherapy instead [for the child] because they were coming from far. But each time I book them, I give them a time. But each time I am ready to have the teletherapy the child might be asleep or would be at a place where it is uncomfortable place to have teletherapy.

HCP

So let me add one more barrier, with the issue of adaptive devices especially if there are no caregivers who really understand or who can be trained on how to retrain the patient on how to use the assistive device it becomes difficult Then unless the patient comes in-person for you to maybe fabricate or measure their assistive device and then train the patient in how to use it.

HCP

### Implementation of Telerehabilitation

#### Stage in the Patient Journey

Service users had a range of opinions on what stage in their rehabilitation journey they preferred to receive telerehabilitation, from the initial stages of diagnosis to using telerehabilitation following a period of in-person rehabilitation.

Initial stages of my condition.

Service user

At the initial stages, you should visit the hospital for physio then later, join the telerehab.

Service user and carer

#### Platform of Choice

WhatsApp was the preferred platform although Microsoft Teams, Zoom, and FaceTime were also used. Ease of use was important with WhatsApp reported to be familiar with options for low data consumption.

Some find zoom cumbersome it’s difficult for them to manage their way through zoom and then having meetings with them, but WhatsApp is just like having a call. I just call you; you see me on the video and then whatever we need to, it’s easier using that platform compared to the other platforms. I used WhatsApp because I think it is easier over here and with data consumption, you know it has the option for you to select low data mode where the streaming is easier for the patient.

HCP

#### Information Governance

Integrating use of personal technology (such as mobile phones) into therapy did cause concern for information security particularly when sharing media such as videos.

I have been thinking about ever since I started working with the clients that I work with. Recently I lost the password to my laptop, and I was thinking there might be a case where a third party has to come in. My phone gets called or any other thing, it’s just confidentiality how is it handled? because a third party will have to come in and help me unlock or do something to my phone Videos, I have a lot of videos of the same person doing mostly I delete them, but I have been thinking about situations where I might forget, or something would happen that another person had access to the videos. So sometimes you know a client may feel uncomfortable sharing videos across, because they don’t know where that would end so that’s one of the barriers I think.

HCP

#### Instructions for Use

Participants felt that further instructions or information was needed to take full advantage of rehabilitation delivered via technology with access to videos requested.

I only had to follow the instructions on the phone.

Service user and carer

I feel like the physiotherapists should be involved in the video demonstrations. There should be visual demonstration videos so the patient can see the physio demonstrating the exercise and imitate it correctly.

Service user and carer

#### Cultural Relevance

It was highlighted that many of the current telerehabilitation exercise videos featured White individuals with instructions in English. To increase the cultural relevance of the materials, videos should be in a local dialect featuring Black individuals completing exercises.

It should be conducted in our local dialect Twi to be specific.

Service user and carer

Language shouldn’t be an issue if we stick to the local dialect. But using English it could be a challenge...another one has to do with the videos where a white person is involved, it makes it difficult for the most clients to understand but it can solve by introducing blacks in such videos.

HCP

## Discussion

### Principal Results

Participants from both focus groups were overall positive in terms of telerehabilitation for people with neurological conditions in Ghana but identified a number of challenges. The conceptual framework for sustainable eHealth in resource-limited countries proposed by Fanta and Pretorius [19], comprising technological, social, economic, and organizational factors, will be used to discuss the findings of the focus groups.

In terms of *technological* factors, one of the main challenges was the lack of a stable internet connection to conduct a telerehabilitation session. This has been identified as one of the main barriers to successful implementation of telerehabilitation in many others studies in LMICs [4,14,15,20], especially in relation to the transfer of images or videos. There was also an appreciation that not all service users had access to a smartphone, as previously reported [5,16]. Interestingly, only 1 therapist raised concerns about the security of service user data particularly when videos of service users completing tasks are sent. In terms of the technology used, this is one of the first studies to report that WhatsApp was the preferred platform for delivering telerehabilitation as it is commonly used in Ghana by both HCPs and service users, so people are familiar with its use and it also has an option for a low data mode. Previous studies of telerehabilitation in LMICs have provided patients with videos to play in a video player [21] or have used Skype [15].

*Social factors* were most commonly raised within the focus groups. Participants overall had positive views on telerehabilitation but also discussed the advantages or preferences of in-person rehabilitation especially with regard to the HCP monitoring their progress. Previous research particularly in relation to patients receiving speech and language therapy also reported good satisfaction with telerehabilitation interventions, but many preferred in-person therapy where there was better eye contact between the patient and the therapist and it was easier to understand facial expression [15,22].

Participants in this study however recognized that there were many barriers to service users accessing in-person rehabilitation. Service users also highlighted the lack of awareness of telerehabilitation services in Ghana.

Like previous studies of telerehabilitation generally [11,13,20] and specifically in relation to LMICs [3,23], participants reported a number of benefits to telerehabilitation especially the convenience, requiring less travel time and reduced travel cost. Some HCPs raised that telerehabilitation improved access to rehabilitation services for patients, especially those who stayed a distance from the clinic. In Ghana, up to 43% of people after stroke access herbal medicines [24], as was raised by 1 person in the focus groups, for which there is little or no efficacy data. Improving access to rehabilitation may reduce the reliance on herbal medicine for some patients.

However, there were also some negative aspects. Not all service users had a smartphone, and furthermore, low levels of literacy and digital skills were barriers to implementing telerehabilitation in line with previous papers [3,4,25]. Although there were examples of some apps being used by therapists, they were not felt to be culturally relevant for the Ghanaian context as they were in English rather than the local dialect and tended to have White people (generally Americans or Europeans) demonstrating the activity, which had the potential to reduce engagement of service users. Odetunde et al [21] developed a telerehabilitation video solution for patients with stroke in Nigeria, delivered both in the local language Yoruba and in English, and this was positively received by participants. To promote uptake and adherence, future development of telerehabilitation interventions should consider the local language and other contextual issues [4,10].

In terms of *economic factors,* as discussed above, not all service users had access to a smartphone, and the financial implications of requiring such a phone and data package were a barrier for many people. Sarfo et al [16] reported that only 35% of their respondents owned a smartphone although 80% had a family member who did. A 2020 household survey of information and communication technology use in Ghana reported that 47.9% of the population had a basic phone and 46.1% had a smartphone; however, in rural areas, 61.3% of people had a basic phone and only 28.1% had a smartphone [26]. However, the figures suggest that family or carers may need to be actively involved for telerehabilitation to be implemented, especially in rural areas of Ghana and in other LMICs. On the positive side, telerehabilitation reduced costs and time required to travel to in-person appointments. In terms of clinical effectiveness, telerehabilitation is variable with some evidence in support of telerehabilitation and other evidence suggesting that it is not superior to conventional care [13,27]. Although there are associated costs, even if it is not superior, the convenience, reach of services, and time-saving aspects would support its further development; however, cost-effectiveness analyses of telerehabilitation for neurological conditions generally and specifically related to resource-limited settings are required [6,9].

*Organizational factors* were generally related to the delivery of the telerehabilitation interventions. Telerehabilitation was felt to be a positive development that could address long waiting times and high demand on services. A number of service users with stroke and PD had used telerehabilitation with examples of telerehabilitation delivery across different allied HCP groups: physiotherapy, occupational therapy, and speech and language therapy. Service users however felt that they would have liked more instructions on how to use telerehabilitation.

HCPs reported using telerehabilitation for undertaking patient assessments, sending information to service users, and receiving videos of patient progress. Neurological rehabilitation often involves intensive therapy with highly repetitive, task-specific exercise to optimize neuroplastic changes in the central nervous system [12]. A novel finding of this study was that therapists reported that telerehabilitation, in this resource-limited setting, allowed more intensive and consistent therapy than would be possible in person and which importantly facilitated increased adherence and improved outcomes. An additional novel finding was that therapists felt that new ways of delivering therapy encouraged them to be more creative in their approach.

The negative aspects considered under organizational factors were the location and timing of telerehabilitation sessions that were arranged in advance; however, when the appointment time came, the service user was not available, or it was not appropriate to complete the session. This often meant that therapists had to have alternative plans in place should that occur. There was also an appreciation that some activities, such as providing assistive devices, required to be done in person.

### Strengths and Limitations

This research had a number of strengths. The views of service users and carers, HCPs, and other stakeholders on telerehabilitation in Ghana were sought directly. Aljabri et al [5] recommended that future research should explore the views of HCPs from different disciplines, which we did, including a range of HCPs such as occupational therapy and speech and language therapy, professions seldom included in the telerehabilitation literature in LMICs—perhaps due to their relatively small numbers compared with, for example, physiotherapy. This is also the first study to include participants with PD from LMICs, although there are previous reviews of telerehabilitation in PD but not in an LMIC context [27]. To be as inclusive as possible, the service user and carer focus group was conducted in the local language. The use of teleconferencing for the stakeholder focus group allowed a wide geographical spread of participants from across Ghana. However, this research also had a number of limitations. Although traveling expenses and refreshments were provided, service users and their carers had to be able to travel to the hospital to take part in the focus group, possibly biasing the sample to a local, urban dwelling and less disabled group. Also, many had had experience of using telerehabilitation, so perhaps they did not represent the views of most people with neurological conditions in Ghana; however, it was important that they were able to share their experiences. Also, none of the study participants resided in a rural setting, thus limiting the transferability of the views captured in this study.

### Conclusions

This is the first study to elicit the views of service users, carers, and HCPs of telerehabilitation for people with neurological conditions in a resource-limited setting of Ghana. The focus group findings overall demonstrated that service users, carers, and HCPs had positive views and experiences of telerehabilitation, especially the convenience and lower cost for service users and the consistency and higher intensity of therapy possible, with some negative aspects including lack of a stable internet connection, cost of phones and data packages, and low levels of literacy. Overall, the findings suggest the need for future research of the clinical and cost-effectiveness of lost cost telerehabilitation interventions for people with neurological conditions, taking into account the local context in Ghana and other LMICs. Telerehabilitation in Ghana is currently not covered within the National Insurance system; however, these findings support the development of telerehabilitation in Ghana with suggestions for future implementation and scale.

## Abbreviations

HCP: health care professional
LMIC: low- to middle-income country
PD: Parkinson disease
